# Supplementary material for: Human LINE-1 retrotransposition requires a metastable coiled coil and a positively charged N-terminus in L1ORF1p
Source: eLife. 2018 Mar 22;7:e34960. doi: 10.7554/eLife.34960 (PMC5940361; doi:10.7554/eLife.34960)
Supplement: Supplementary file 1. — Mammalian L1ORF1p sequences are from Boissinot and Sookdeo (Boissinot and Sookdeo, 2016) apart from the human sequence (NCBI accession L19088.1) (Dombroski et al., 1993), the mouse sequences (Sookdeo et al., 2013) and the megabat sequence (NCBI accession KF796623.1) (Yang et al., 2014). The conserved and alignable portions of the proteins are in bold letters (Khazina et al., 2011), and the coiled coil domains are listed as heptads as in Figure 1C. Regular heptads are shaded grey and black and non-heptad interruptions are shaded in magenta, corresponding to the colors in Figure 1—figure supplement 1B. Similarly, the NTRs are light grey with the positively charged N-terminal peptides in blue, the RRM domains are in red and the CTDs are in cyan. Coiled coil propensity was calculated using PCoils (Alva et al., 2016; Lupas, 1996) and used to assign heptads by manual inspection of results from PCoils. Remaining ambiguities in the precise assignment of non-heptad interruptions require finer sequence sampling or experimental validation to be resolved. [file elife-34960-supp1.docx]

**Supplementary file 1**

**Mammalian L1ORF1p sequences used in Figure 1 – figure supplement 1B**

**>HUMAN**

MGKKQNRKTGNSKTQSASPPPKERSSSPATEQSWMENDFDELREEGFRRSN

YSELRED

IQTKGKE

VENFEKN

LEECITR

ITNTEKC

LKEL

-MELKTK

ARELREE

**CRSLRSR**

**CDQLEER**

**VSAMEDE**

**MNEMKRE**

**GKFREKR**

**IKRNEQS**

**LQEIWDY**

**VKRP**

**NLRLIGVPESDVENGTKLENTLQDIIQENFPNLARQANVQIQEIQRTPQRYSSRRATPRHIIVRFTKVEMKEKMLRAAREKGRVTLKGKPIRLTVD**

**LSAETLQARREWGPIFNILKEKNFQPRISYPAKLSFISEGEIKYFIDKQMLRDFVTTRPALKELLKEAL**

NMERNNRYQPLQNHAKM

**>LEMUR**

MGRNQRKNSGNMKNQTENTPPRRSTSPLETDTDQNQATNMTEKEFRMWI

IRTLTQL

QQQLNNQ

HQENTKN

LQEM

EQRFNKE

IDTVKKT

LTEV

-LEMKNQ

LRELQNT

**VESLKNR**

**VDQAEER**

**ISELEDN**

**TFQLNKS**

**VTEIQQR**

**NKRKDQS**

**LQELWDY**

**VKKP**

**NVRVIGLAEGEEDNTQGLDKLFEDIIEENFPGLAQNLDIQVQEAQRTPGRFNANRKTSRHAVIRLTKVSTKEALLRAVRQKKQVTYKGKPIRITSD**

**FSNETLQARRDWGPILTLLKQNNAQPRILFPAKLSFVYEGEIKTFSDKQRLREFTKTRPALQEVLKTAL**

RTEHHNNNPRI

**>MOUSE_A1**

MAKGKRKNPTNRNQDHSPSSERSTPTPPSPGHPNTTENLDPDLKTFLMMMIEDIKKDF

HKSLKDL

QESTAKE

LQALKEK

QENTAKQ

VMEMNKT

ILELKGE

VDTIKKT

QSEA

TLEIETL

GKRSGTI

**DASISNR**

**IQEMEER**

**ISGAEDS**

**IENIDTT**

**VKENTKC**

**KRILTQN**

**IQVIQDT**

**MRRP**

**NLRIIGIDENEDFQLKGPANIFNKIIEENFPNIKKEMPMIIQEAYRTPNRLDQKRNSSRHIIIRTTNALNKDRILKAVREKGQVTYKGRPIRITPD**

**FSPETMKARRAWTDVIQTLREHKCQPRLLYPAKLSITIDGETKVFHDKTKFTQYLSTNPALQRIITEKK**

QYKDGNHALEQPRK

**>MOUSE_Tf1**

MAKGKRRNLTNRNQDHSPSPEPSTPTSPSPGNPNTPENLDLDLKAYLMMMVEDIKKDF

NKSLKEI

QENTAKE

LQVLKEK

QENTIKQ

VEVLTEK

EEKTYKQ

VMEMNKT

ILDLKRE

VDTIKKT

QSEA

TLEIETL

GKKSGTI

**DLSISNR**

**IQEMEER**

**ISGAEDS**

**IENIGTT**

**IKENGKC**

**KKILTQN**

**IQEIQDT**

**IRRP**

**NVRIIGVDENEDFQLKGPANIFNKIIEENFPNLKNEMHMNIQEAYRTPNRLDQKRNSSRHIIIRTSNALNKDRILKAVREKGQVTYKGKPIRITPD**

**FSPETMKARRAWTDVIQTLREHKLQPRLLYPAKLSIIIEGETKVFHDKTKFTHYLSTNPALQRIITEKN**

QYKNGNNALEKTRR

**>MOUSE_Gf1**

MAKGKRKNPTNRSQDHSPSSEPRTPTSPNPGHPNTPEKVDLDLKAYLMMMVEDIKKEF

XNSLKEI

QENTAKE

LQVLKEK

QENTAKE

LQVLKEK

QENTTKQ

VEVLIEK

QENTSKQ

VMEMNKT

ILDLKRE

VDTIKKT

QSEA

TLEIETL

GKKSGTI

**DASISNR**

**IQEMEER**

**ISGAEDS**

**IENIGTT**

**IKENGKC**

**KKILTQN**

**IQEIQDT**

**MRRP**

**NLRIIGVDENEDFQLKGPANIFNKIIEENFPNLKKEMPMNIQEAYRTPNRLDQKRNSSRHIIIRTPNALNKDRILKAVREKGQVTYKGKPIRITPD**

**FSPETMKARRAWXDVIQTLREXKCQPRLLYPAKLSITIDGETKVFHDKTKFTHYLSTNPALQRIITEKX**

QYKDGNHALEKTRR

**>RAT**

MARGKRRNPSNRNQDYMPSSEPNSPTKTNMEYPNTPEKQDLVSKSYLIMMLEDFRKD

LNTLREA

QENINKQ

VKAYREE

SQKS

LKEF

QENTIKQ

LKELKME

IEAIKKE

HMET

TLDIENQ

KKRQGAV

**DTSFTNR**

**IQEMEER**

**ISGAEDS**

**IEIIDST**

**VKDNVKR**

**KKLLVQN**

**IQEIQDS**

**MRRS**

**NLRIIGIEESEDSQLKGPVNIFNKIIEENFPNLKKEIPIDIQEAYRTPNRLDQKRNTSRHIIVKTPNAQNKERILKAVREKGQVTYKGRPIRITPD**

**FSPETMKARRSWTDVIQTLREHKCQPRLLYPAKLSINIDGETKIFHDKTKFTQYLSTNPALQRIINGKA**

QHKEASYTLEEARN

**>RABBIT**

MPNNKRKNRGNKNKEVTMTPSNEKDTPIQDYEDDDIEEMQEADLKKL

IRTLRSS

QKQILEL

QKSLMDK

IENLSRE

NEILRRN

QNETKQL

VQQETVI

VTEVKNS

IDQMKNT

**IESLTNR**

**MGEAEER**

**ISDLEDR**

**EQERIQS**

**DQRKEEE**

**IRNLKHI**

**VGNLQDT**

**IKKP**

**NIRVLGVPEGMEREKGLEGLFSEILAENFPGLEKDREILVQEAHRTPNKHDQKRSSPRHVVIKLSTVKHKEKILKCAREKRQITLRGSPIRLTAD**

**FSSETLQARREWRDIAQVLREKNCQPRILYPAKLSFVNEGEIKTFHSKQKLKEFVATRPALQKMLKDVL**

HTETQKHGHQYERR

**>PIG**

MKKLRNHPQSNQQENSPKTVNNETDLCSLTDLEFKREI

VKILKEL

REDMNSN

ADTLRKE

LENIRRS

QEKLEHS

FAEMQTE

**LGAVKTR**

**MNNAEER**

**ISDMEDR**

**IMEITQS**

**GQQTENR**

**IKKLESN**

**IRDLWDN**

**IKRA**

**NLRIIGIPEGVEKDKGMENIFEEIIAGNFPNLKDTGFKIQEAQRAPNKLNPNRPTPRHIIIKMAKVSDKERILKAAREKQNVTYKGTPIRISAD**

**FSTETLQARREWQEIFKVLKGKNMQPRILYPARISFKIEGEIKIFPNKQKLKEYSNTKPRLKEILKGLL**

**>COW**

MKRQRNTQQIKEQDKCPPNQTKEEEIGNLPDKEFRIMI

VKLIQNL

ETKMESQ

INSLETR

IEKM

QERFNKD

LEEIKKS

QYIMNNA

ISEIKNT

**LEATNSR**

**ITEAEDR**

**ISELEDR**

**MVEINES**

**ERIKEKR**

**IKRNEDN**

**LRDLQDN**

**IKRY**

**NIRIIGVPEEEDKKKDHEKILEEIIVENFPKMGKEIITQVQETQRVPNRINPRRNTPRHILIKLTKIKHKEQILKAAREKQQITHKGIPIRITAD**

**LSIETLQARREWQDILKMMKENNLQPRLLYPARISFKYEGEIKSFSDKQKLREFCTTKPALQQILKDIL**

**>HORSE**

MRRHKSTSSSNMKKYIKSPEQKESNKYTENNPKENEIYNLNDDDFKTAI

IKILTEL

RENSDRQ

LNEFRSY

VTKEFDT

IKKNQTE

ILEMKNT

IEEIKKN

**LDALNSR**

**ADNMEER**

**ISNLEDG**

**NIELLQA**

**EEEREAR**

**LKRNEET**

**LRELSDT**

**IRRC**

**NVRIIGIPEGEEKEKGAENLFKEIMAENFPNLVREMDLQVTEANRSPNFINARRPTPRHIVVKLAKVNDKEKILRTARQKKLTYKGTPIRLSAD**

**FSAETLQARREWNDIFKNLKDKNLQPRILYPAKISFKYDGEIKTFPDKQKLREFIATKPPLQEILRKTL**

IPEKSKKGKGLQNQEQRR

**>DOG**

MTRRKTSPQKKESETVLSPTELQNLDYNSMSESQFRSTI

IQLLVAL

EKSI

KDSRDFM

TAEFRAN

QAEIKNQ

LNEMQSK

**LEVLTTR**

**VNEVEER**

**VSDLEDK**

**LIAKRET**

**EEKRDKQ**

**LKDHEDR**

**LREINDS**

**LRKK**

**NLRLIGVPEGAERDRGPEYVFEQILAENFPNLGRETGIQIQEIERSPPKINKNRSTPRHLIVKLANSKDKEKILKAARDKKSLTFMGRSIRVTAD**

**LSTETWQARKGWQDIFRVLNEKNMQPRILYPARLSFKMEGEIKSFQDRQQLKEYVTSKPALQEILRGPL**

KIPL

**>PANDA**

MTRRRSPPQQRKDNESVASATELASATELIHTDVSQLSEMEFRATM

VKMMSKL

EKSI

RESV

AENIESL

RAEMRAN

LTEIKNS

VGQIQSK

**LEALTAR**

**VTEAEER**

**VSELEDG**

**LVEEKTK**

**IEAGLKK**

**IHAHECR**

**LREITDS**

**MKRS**

**NVRIIGIPEGVEKNRGLEEIFEQIVAENFPNLARETSIRVQEAERTPSKLNQDKPTPRHVIVQFANIRSKDTVLKAARAKKFLTYQGKGIRITSD**

**LSTETWNERKAWGGIFKALSEKNMQPRILYPAKLSFRIDGEIKTFQNRQSLTNFVTTKPALQEILRGAL**

**>MEGABAT**

MPRRRNSPLNTMNNQGDKASQKEREKSPENKLTDIEICDLNDREFRMVL

LKKLSEF

RNESYQQ

FQELKKQ

LDEQSKE

IETLKKN

RIEL

-LEIKNT

LQELKNE

**IASLGNR**

**VDQMEER**

**ISDIEDR**

**NLEINQK**

**EEERNRR**

**MKNNERE**

**IQELADT**

**IRRG**

**NIRIMGIIEGEEKEQGLESIFRQIVDENFPNLRNELELGIQEVNRTPNYLNPKRPSPRHIVLKLSKINDKDRILRAAREKKTVTYKGKPIRLSSD**

**FSAQTLQARKEWNQIFKLLSERNYQPRIMYPAKLSFRYEGEIKTFPDIQKLREFSTTRPALQEILKGVS**

SPKTK

**>ELEPHANT**

MARRQQSISNHLKKQTMTASPTPQTKESKSFPNEDTILELSDTEYKKLI

YRMLKDI

TNEIRIT

AEKAKEH

TDKT

VEELKKI

IQEHSGK

INKLQES

IERQHVE

IQKINNK

ITELDNA

**IGSQRSR**

**LEQLECR**

**LGHLEDQ**

**GINTNIA**

**EKKSDKR**

**IKKNEET**

**LRIMWDS**

**IKKD**

**NLRVIGVPEQGGGTENTEKIVEELLTQNFPDIMKDERISIQDAHRTPFKIDPKRKTPRHIIIKLTKTKDKQKILKAAREKRKVSFKGESIRISSD**

**YSAETMQARREWDDIYRTLKEKNCQPRIIYPAKLSLKYEGEIKIFTDKHKFREFAKTKPKLQEILKDIV**

WSENQ

**>HYRAX**

MGPKKQQTIVAYKQSLMASEGSQTKQPRSFPDEETFMKASGSEYKLII

YRALLDL

IQNA

-AEQNEK

LSKLFDL

MEHQYSE

IQQIKKK

IKDIETS

**MQSQENR**

**MEQMEKR**

**ISDLEDR**

**ATDASVY**

**EQKSEKR**

**NKKNEET**

**LRSMWDS**

**IKRN**

**NLRLIGVPEQGETSENTESIVAELLKENFPEIMKEENIDISDAYRTPPNIDLKRKTPRHIIIKLNKMKHKQQLLKAARLKAKLTFRGKPCRLSSD**

**FSAETMLARRQWHDTFKVLKEKNFQPRIIYPAKLSFKYENEIKTFPDKQKLRDFVKTKPKLQEILKEVL**

GFGLEERNH

**>ARMADILLO**

MMTRQQQKSTNQTSNQENMAESNEQTKNQEGEQNFAQV

MKDLRTF

ITDK

FNEVKEE

VNNMKTT

LGGEIAD

IRKKITD

MMGMNTT

VQEIKNT

**LAANISR**

**LEEAEQR**

**ISDVEDS**

**TSEIKQI**

**VELVDKK**

**IEKIQLG**

**LRDLNDN**

**AKRS**

**NIGIIGIPEGEEKGKGSEGVLQEIMAENFPNLLKETDVHIQEAQRTPIVINPNRPTPRHILVKLSNAQDKEKILKAAREKKTITYKGSSIRLSAD**

**FSSETMEARRQWYDIVKVLKEKNFQPRILYPAKLAFKNDGEFKIFTDKQKLKEYANKKPPLQEILKGVL**

QEERKKQDRQSWRRV
