# Supplementary material for: Human LINE-1 retrotransposition requires a metastable coiled coil and a positively charged N-terminus in L1ORF1p
Source: eLife. 2018 Mar 22;7:e34960. doi: 10.7554/eLife.34960 (PMC5940361; doi:10.7554/eLife.34960)
Supplement: Supplementary file 2. — Primate L1ORF1p sequences in Figure 1—figure supplement 2 are reconstructed consensus sequences with 60% residue identity in the alignments of the listed accessions. [file elife-34960-supp2.docx]

**Supplementary file 2**

**Individual accessions for primate L1ORF1p**

**Pan_troglodytes:**

>gi|291061363:62023525-62024538

>gi|291061364:85754769-85755782

>gi|291061376:c152862775-152863788

>gi|291061374:c227199386-227200399

>gi|319999821:c113968167-113969180

>gi|291061371:c58696897-58697910

>gi|291061368:c16554545-16555558

>gi|678170650:c31752112-31753125

>gi|291061372:141489494-141490507

>gi|291061370:c161916994-161918013

**Pongo_abelii:**

>gb|AC212868.3|:c58811-59824

>gb|AC205915.3|:174912-175925

>gb|AC188113.1|:66422-67435

>gb|AC198052.3|c:110151-111164

>gb|AC187577.3|:c9849-10862

>gb|AC199446.3|:c21834-22847

>gb|AC200336.2|:c50932-51945

>gb|AC210376.3|:112104-113117

>gb|AC200628.3|:c1507-2520

>gb|AC205913.3|:170380-171393

**Nomascus_leucogenys:**

>gi|98986042:52153-53112

>gb|AC198102.2|:c114210-113197

>gb|AC208963.3|:c141433-140420

>gb|AC198097.2|:3262-4275

>gi|112182807:125023-126036

>gb|AC214972.4|:96456-97469

>gi|110783224:c150906-149893

>gb|AC215319.3|:11834-12847

>gi|123711080:124102-125115

>gi|110566930:159037-160050

**Macaca_mulatta:**

>gb|AC215984.2|:c59650-58637

>gb|AC198952.3|:c179125-178112

>gb|AC214621.4|:c57074-56061

>gb|AC201631.4|:c146571-145558

>gb|AC214205.7|:140135-141148

>gb|AC199580.6|:119382-120395

>gb|AC205191.3|:101140-102153

>gb|KT332914.1|:c4467-3454

>gb|KT332833.1|:c23318-22305

>gb|KJ489617.1|:c24569-23556

**Papio_anubis:**

>gb|AC091778.13|:145055-146068

>gb|AC113268.8|:13294-14307

>gb|AC099742.2|:158914-159927

>gb|AC098698.3|:106686-107699

>gb|AC136143.4|:c127698-126685

>gb|AC173452.3|:c83937-82924

>gb|AC139345.8|:52119-53129

>gb|AC089990.3|:c116932-115919

>gb|AC091381.3|:c20780-19767

>gb|AC091671.28|:127541-128554

**Colobus_guereza:**

>gi|80751225|gb|AC160873.4|:120422-121435

>gi|75905564|gb|AC163238.3|:192822-193835

>gi|80751224|gb|AC153080.4|:80455-81468

>gi|80751223|gb|AC153740.3|:190197-191210
