# Supplementary material for: Human LINE-1 retrotransposition requires a metastable coiled coil and a positively charged N-terminus in L1ORF1p
Source: eLife. 2018 Mar 22;7:e34960. doi: 10.7554/eLife.34960 (PMC5940361; doi:10.7554/eLife.34960)
Supplement: Supplementary file 3. [file elife-34960-supp3.docx]

**Supplementary file 3**

**Plasmid constructs based on pJM101 (Moran et al., 1996; Sassaman et al., 1997)**

| **Protein expression (*E.coli*)** | |
| --- | --- |
| **Construct name** | **Description** |
| pET15b-hL1ORF1p-NTR(H6) | A fragment (residues 1-51) of human LINE-1 ORF1 coding sequence from pJM101 inserted into pET15b (Novagen) for bacterial expression and to generate a non-cleavable 6xHis tag at the C-terminus. |
| pET15b-hL1ORF1p(DD)-NTR(H6) | A fragment (residues 1-51) of human LINE-1 ORF1 coding sequence from pJM101 inserted into pET15b (Novagen) for bacterial expression and to generate a non-cleavable 6xHis tag at the C-terminus. (Phospho-mimicking point mutations: S18D, S27D) |
| pnEA-pH-hL1ORF1p-Δcons | A fragment (residues 1-103) of human LINE-1 ORF1 coding sequence from pJM101 inserted into pnEA-pH (Diebold et al., 2011) for bacterial expression and to generate a cleavable 6xHis tag at the N-terminus. |
| pnEA-pH-hL1ORF1p(AII)-cc | A fragment (residues 53-152) of human LINE-1 ORF1 coding sequence from pJM101 inserted into pnEA-pH (Diebold et al., 2011) for bacterial expression and to generate a cleavable 6xHis tag at the N-terminus (Engineered point mutations: M121A, M125I, M128I) (Khazina et al., 2011). |
| pnEA-pH-hL1ORF1p(AII)-cc(Δ91-93) | A fragment (residues 53-152) of human LINE-1 ORF1 coding sequence from pJM101 inserted into pnEA-pH (Diebold et al., 2011) for bacterial expression and to generate a cleavable 6xHis tag at the N-terminus with a stammer deletion (Engineered point mutations: M121A, M125I, M128I) (Khazina et al., 2011). |
| pnEA-pH-hL1ORF1p(AII)-ΔNTR | A fragment (residues 53-338) of human LINE-1 ORF1 coding sequence from pJM101 inserted into pnEA-pH (Diebold et al., 2011) for bacterial expression and to generate a cleavable 6xHis tag at the N-terminus (Engineered point mutations: M121A, M125I, M128I) (Khazina et al., 2011). |

| **Retrotransposition assay** | |
| --- | --- |
| **Construct name** | **Description** |
| pCEP4-L1.3 | Human LINE-1 coding sequence in pJM101 for expression in Hela cells. |
| pCEP4-L1.3-D702A | Human LINE-1 coding sequence in pJM101 for expression in Hela cells with a mutation in the active site of reverse transcriptase encoded by ORF2p. |
| pCEP4-L1.3-ORF1p-Δ2-6 | Human LINE-1 coding sequence in pJM101 for expression in Hela cells with a deletion in ORF1p. |
| pCEP4-L1.3-ORF1p-Δ2-11 | Human LINE-1 coding sequence in pJM101 for expression in Hela cells with a deletion in ORF1p. |
| pCEP4-L1.3-ORF1p-Δ2-16 | Human LINE-1 coding sequence in pJM101 for expression in Hela cells with a deletion in ORF1p. |
| pCEP4-L1.3-ORF1p-Δ2-21 | Human LINE-1 coding sequence in pJM101 for expression in Hela cells with a deletion in ORF1p. |
| pCEP4-L1.3-ORF1p-Δ2-28 | Human LINE-1 coding sequence in pJM101 for expression in Hela cells with a deletion in ORF1p. |
| pCEP4-L1.3-ORF1p-Δ2-51 | Human LINE-1 coding sequence in pJM101 for expression in Hela cells with a deletion in ORF1p. |
| pCEP4-L1.3-ORF1p-Δ2-88 | Human LINE-1 coding sequence in pJM101 for expression in Hela cells with a deletion in ORF1p. |
| pCEP4-L1.3-ORF1p-Δ29-51 | Human LINE-1 coding sequence in pJM101 for expression in Hela cells with a deletion in ORF1p. |
| pCEP4-L1.3-ORF1p-Δ52-65 | Human LINE-1 coding sequence in pJM101 for expression in Hela cells with a deletion in ORF1p. |
| pCEP4-L1.3-ORF1p-Δ52-89 | Human LINE-1 coding sequence in pJM101 for expression in Hela cells with a deletion in ORF1p. |
| pCEP4-L1.3-ORF1p-Δ52-103 | Human LINE-1 coding sequence in pJM101 for expression in Hela cells with a deletion in ORF1p. |
| pCEP4-L1.3-ORF1p-Δ91-93 | Human LINE-1 coding sequence in pJM101 for expression in Hela cells with a deletion in ORF1p. |
| pCEP4-L1.3-ORF1p-G132I/R135I/N142I | Human LINE-1 coding sequence in pJM101 for expression in Hela cells with point mutations in ORF1p. |
| pCEP4-L1.3-ORF1p-G132I/R135I | Human LINE-1 coding sequence in pJM101 for expression in Hela cells with point mutations in ORF1p. |
| pCEP4-L1.3-ORF1p-N142I | Human LINE-1 coding sequence in pJM101 for expression in Hela cells with a point mutation in ORF1p. |
| pCEP4-L1.3-ORF1p-R135I | Human LINE-1 coding sequence in pJM101 for expression in Hela cells with a point mutation in ORF1p. |
| pCEP4-L1.3-ORF1p-R135N | Human LINE-1 coding sequence in pJM101 for expression in Hela cells with a point mutation in ORF1p. |
| pCEP4-L1.3-ORF1p-G132V | Human LINE-1 coding sequence in pJM101 for expression in Hela cells with a point mutation in ORF1p. |
| pCEP4-L1.3-ORF1p-L93N/L100N | Human LINE-1 coding sequence in pJM101 for expression in Hela cells with point mutations in ORF1p. |
| pCEP4-L1.3-ORF1p-L107N/L114N | Human LINE-1 coding sequence in pJM101 for expression in Hela cells with point mutations in ORF1p. |
| pCEP4-L1.3-ORF1p-C104I/C111I | Human LINE-1 coding sequence in pJM101 for expression in Hela cells with point mutations in ORF1p. |
| pCEP4-L1.3-ORF1p-C86S | Human LINE-1 coding sequence in pJM101 for expression in Hela cells with a point mutation in ORF1p. |
| pCEP4-L1.3-ORF1p-R48A | Human LINE-1 coding sequence in pJM101 for expression in Hela cells with a point mutation in ORF1p. |
| pCEP4-L1.3-ORF1p-G2A/K3A | Human LINE-1 coding sequence in pJM101 for expression in Hela cells with point mutations in ORF1p. |
| pCEP4-L1.3-ORF1p-G2A | Human LINE-1 coding sequence in pJM101 for expression in Hela cells with a point mutation in ORF1p. |
| pCEP4-L1.3-ORF1p-K3A | Human LINE-1 coding sequence in pJM101 for expression in Hela cells with a point mutation in ORF1p. |
| pCEP4-L1.3-ORF1p-K4A | Human LINE-1 coding sequence in pJM101 for expression in Hela cells with a point mutation in ORF1p. |
| pCEP4-L1.3-ORF1p-G2R | Human LINE-1 coding sequence in pJM101 for expression in Hela cells with a point mutation in ORF1p. |
| pCEP4-L1.3-ORF1p-K3R | Human LINE-1 coding sequence in pJM101 for expression in Hela cells with a point mutation in ORF1p. |
| pCEP4-L1.3-ORF1p-K13A | Human LINE-1 coding sequence in pJM101 for expression in Hela cells with a point mutation in ORF1p. |
| pCEP4-L1.3-ORF1p-myr | Human LINE-1 coding sequence in pJM101 for expression in Hela cells with the N-terminus of ORF1p mutated to a canonical myristoylation sequence. |
| pCIneo-RLuc-ΔSV40neo | A plasmid expressing *R. reniformis* luciferase in mammalian cells (Lazzaretti et al., 2009) with a deletion of SV40 promoter and of the gene encoding resistance to neomycin. |

| **Protein expression (mammalian cells)** | |
| --- | --- |
| **Construct name** | **Description** |
| pCEP4-L1.3-ORF1p-HA | Human LINE-1 coding sequence in pJM101 for expression in Hela cells with an HA-tag on the C-terminus of ORF1p. |
| pCEP4-L1.3-ORF1p-Δ2-6-HA | Human LINE-1 coding sequence in pJM101 for expression in Hela cells with a deletion in ORF1p and an HA-tag on the C-terminus of ORF1p. |
| pCEP4-L1.3-ORF1p-Δ2-11-HA | Human LINE-1 coding sequence in pJM101 for expression in Hela cells with a deletion in ORF1p and an HA-tag on the C-terminus of ORF1p. |
| pCEP4-L1.3-ORF1p-Δ2-16-HA | Human LINE-1 coding sequence in pJM101 for expression in Hela cells with a deletion in ORF1p and an HA-tag on the C-terminus of ORF1p. |
| pCEP4-L1.3-ORF1p-Δ2-21-HA | Human LINE-1 coding sequence in pJM101 for expression in Hela cells with a deletion in ORF1p and an HA-tag on the C-terminus of ORF1p. |
| pCEP4-L1.3-ORF1p-Δ2-28-HA | Human LINE-1 coding sequence in pJM101 for expression in Hela cells with a deletion in ORF1p and an HA-tag on the C-terminus of ORF1p. |
| pCEP4-L1.3-ORF1p-Δ2-51-HA | Human LINE-1 coding sequence in pJM101 for expression in Hela cells with a deletion in ORF1p and an HA-tag on the C-terminus of ORF1p. |
| pCEP4-L1.3-ORF1p-Δ2-88-HA | Human LINE-1 coding sequence in pJM101 for expression in Hela cells with a deletion in ORF1p and an HA-tag on the C-terminus of ORF1p. |
| pCEP4-L1.3-ORF1p-Δ29-51-HA | Human LINE-1 coding sequence in pJM101 for expression in Hela cells with a deletion in ORF1p and an HA-tag on the C-terminus of ORF1p. |
| pCEP4-L1.3-ORF1p-Δ52-65-HA | Human LINE-1 coding sequence in pJM101 for expression in Hela cells with a deletion in ORF1p and an HA-tag on the C-terminus of ORF1p. |
| pCEP4-L1.3-ORF1p-Δ52-89-HA | Human LINE-1 coding sequence in pJM101 for expression in Hela cells with a deletion in ORF1p and an HA-tag on the C-terminus of ORF1p. |
| pCEP4-L1.3-ORF1p-Δ52-103-HA | Human LINE-1 coding sequence in pJM101 for expression in Hela cells with a deletion in ORF1p and an HA-tag on the C-terminus of ORF1p. |
| pCEP4-L1.3-ORF1p-Δ91-93-HA | Human LINE-1 coding sequence in pJM101 for expression in Hela cells with a deletion in ORF1p and an HA-tag on the C-terminus of ORF1p. |
| pCEP4-L1.3-ORF1p-G132I/R135I/N142I-HA | Human LINE-1 coding sequence in pJM101 for expression in Hela cells with point mutations in ORF1p and an HA-tag on the C-terminus of ORF1p. |
| pCEP4-L1.3-ORF1p-G132I/R135I-HA | Human LINE-1 coding sequence in pJM101 for expression in Hela cells with point mutations in ORF1p and an HA-tag on the C-terminus of ORF1p. |
| pCEP4-L1.3-ORF1p-N142I-HA | Human LINE-1 coding sequence in pJM101 for expression in Hela cells with a point mutation in ORF1p and an HA-tag on the C-terminus of ORF1p. |
| pCEP4-L1.3-ORF1p-R135I-HA | Human LINE-1 coding sequence in pJM101 for expression in Hela cells with a point mutation in ORF1p and an HA-tag on the C-terminus of ORF1p. |
| pCEP4-L1.3-ORF1p-R135N-HA | Human LINE-1 coding sequence in pJM101 for expression in Hela cells with a point mutation in ORF1p and an HA-tag on the C-terminus of ORF1p. |
| pCEP4-L1.3-ORF1p-G132V-HA | Human LINE-1 coding sequence in pJM101 for expression in Hela cells with a point mutation in ORF1p and an HA-tag on the C-terminus of ORF1p. |
| pCEP4-L1.3-ORF1p-L93N/L100N-HA | Human LINE-1 coding sequence in pJM101 for expression in Hela cells with point mutations in ORF1p and an HA-tag on the C-terminus of ORF1p. |
| pCEP4-L1.3-ORF1p-L107N/L114N-HA | Human LINE-1 coding sequence in pJM101 for expression in Hela cells with point mutations in ORF1p and an HA-tag on the C-terminus of ORF1p. |
| pCEP4-L1.3-ORF1p-C104I/C111I-HA | Human LINE-1 coding sequence in pJM101 for expression in Hela cells with point mutations in ORF1p and an HA-tag on the C-terminus of ORF1p. |
| pCEP4-L1.3-ORF1p-C86S-HA | Human LINE-1 coding sequence in pJM101 for expression in Hela cells with a point mutation in ORF1p and an HA-tag on the C-terminus of ORF1p. |
| pCEP4-L1.3-ORF1p-R48A-HA | Human LINE-1 coding sequence in pJM101 for expression in Hela cells with a point mutation in ORF1p and an HA-tag on the C-terminus of ORF1p. |
| pCEP4-L1.3-ORF1p-G2A/K3A-HA | Human LINE-1 coding sequence in pJM101 for expression in Hela cells with point mutations in ORF1p and an HA-tag on the C-terminus of ORF1p. |
| pCEP4-L1.3-ORF1p-G2A-HA | Human LINE-1 coding sequence in pJM101 for expression in Hela cells with a point mutation in ORF1p and an HA-tag on the C-terminus of ORF1p. |
| pCEP4-L1.3-ORF1p-K3A-HA | Human LINE-1 coding sequence in pJM101 for expression in Hela cells with a point mutation in ORF1p and an HA-tag on the C-terminus of ORF1p. |
| pCEP4-L1.3-ORF1p-K4A-HA | Human LINE-1 coding sequence in pJM101 for expression in Hela cells with a point mutation in ORF1p and an HA-tag on the C-terminus of ORF1p. |
| pCEP4-L1.3-ORF1p-G2R-HA | Human LINE-1 coding sequence in pJM101 for expression in Hela cells with a point mutation in ORF1p and an HA-tag on the C-terminus of ORF1p. |
| pCEP4-L1.3-ORF1p-K3R-HA | Human LINE-1 coding sequence in pJM101 for expression in Hela cells with a point mutation in ORF1p and an HA-tag on the C-terminus of ORF1p. |
| pCEP4-L1.3-ORF1p-K13A-HA | Human LINE-1 coding sequence in pJM101 for expression in Hela cells with a point mutation in ORF1p and an HA-tag on the C-terminus of ORF1p. |
| pCEP4-L1.3-ORF1p-myr-HA | Human LINE-1 coding sequence in pJM101 for expression in Hela cells with the N-terminus of ORF1p mutated to a canonical myristoylation sequence and an HA-tag on the C-terminus of ORF1p. |
| p7-EGFP-C1-MBP | A plasmid expressing EGFP-MBP fusion protein in mammalian cells (Lazzaretti et al., 2009). |
| pcDNA3.1 | Vector for mammalian gene expression (Invitrogen). |
